# Measuring the successes and deficiencies of constant pH molecular dynamics: A blind prediction study

**DOI:** 10.1002/prot.23136

**Published:** 2011-07-22

**Authors:** Sarah L Williams, Patrick G Blachly, J Andrew McCammon

**Affiliations:** 1 Department of Chemistry & Biochemistry, University of California San DiegoLa Jolla, California 92093-0365; 2 Center for Theoretical Biological Physics, University of California San DiegoLa Jolla, California 92093; 3 Howard Hughes Medical Institute, University of California San DiegoLa Jolla, California 92093-0365; 4 Department of Pharmacology, University of California San DiegoLa Jolla, California 92093-0365

**Keywords:** constant pH molecular dynamics (CpHMD), p*K*_a_ prediction, implicit salvation, Monte Carlo

## Abstract

A constant pH molecular dynamics method has been used in the blind prediction of p*K*_a_ values of titratable residues in wild type and mutated structures of the Staphylococcal nuclease (SNase) protein. The predicted values have been subsequently compared to experimental values provided by the laboratory of García-Moreno. CpHMD performs well in predicting the p*K*_a_ of solvent-exposed residues. For residues in the protein interior, the CpHMD method encounters some difficulties in reaching convergence and predicting the p*K*_a_ values for residues having strong interactions with neighboring residues. These results show the need to accurately and sufficiently sample conformational space in order to obtain p*K*_a_ values consistent with experimental results.

## INTRODUCTION

It is well established that the structure and function of a protein are highly dependent on the pH of its surrounding environment. The p*K*_a_ of a titratable residue, which is heavily influenced by interactions with neighboring residues within a protein, governs the protonation state of that residue for a given solution pH. Changes in protonation state within a protein manifest as alterations to the charge distribution of the titratable residue, influencing the electrostatics of the protein environment. Protonation equilibria are thus closely linked with protein conformation, evidence of which is the sensitivity of proteins to denaturation at extreme pH.

The interplay between protonation state and protein conformation is not accounted for in conventional molecular dynamics (MD) simulations. Currently, these simulations employ fixed, predetermined protonation states for titratable residues, which are generally chosen according to the p*K*_a_ value of the respective residue when isolated in solution. This method of protonation state assignment can be a severe approximation, as the p*K*_a_ values of titratable residues are frequently shifted from that of the isolated residue in solution. Furthermore, protonation states are not constant, but rather exist in equilibria, subject to the changing electrostatic environment surrounding the titratable group. Therefore, incorporating pH as an input variable in MD simulations is highly desirable, as it would allow a more accurate study of pH-coupled protein dynamics, such as ligand binding and protein folding.

Over the past few decades, a number of theoretical methods have been developed to try to accurately determine the protonation states of titratable residues in proteins. One class of methods utilizes static protein structures and employs a Poisson–Boltzmann approach for the calculation of electrostatics.^1^–^3^ However, the use of static structures is thought to be a major contributor to discrepancies observed in the calculation of p*K*_a_ shifts, as the conformational changes in the protein induced by change in residue protonation state are not taken into account. More recently, these methods have been improved by including descriptions of conformational variability, with adaptations to account for dielectric heterogeneity^4^, ^5^ and inclusion of conformational flexibility.^6^–^9^ Notably, Warshel and coworkers were the first to employ MD methods to improve calculation of p*K*_a_ values in proteins, with their electrostatic protein dipoles Langevin dipoles (PDLD) model.^10^ Other groups have incorporated MD and QM/MM methods coupled with free energy perturbation techniques for p*K*_a_ calculations.^11^–^13^ A drawback of these techniques is their high reliance on the resolution of the input structure, which renders these methods incapable of calculating p*K*_a_ shifts where protonation is accompanied by large conformational change.

Another class of methods incorporates the important coupling of conformation and protonation state through the use of computational simulations that employ pH as an external thermodynamic parameter.^14^–^26^ These methods are often described as either continuous or discrete constant pH methods, contingent on how titratable protons are considered within the simulation. The former treats protonation state as a continuous titration parameter that advances simultaneously with the atomic coordinates of the system.^19^–^21^ Originally, the implementation of this method used a mean-field approximation, and protonation sites could exist as fractionally occupied. More recently, Lee *et al*., have developed methods to overcome issues with fractional protonation states, using λ-dynamics with an artificial titration barrier to discourage fractional protonation.^22^ Extensions to the work of Lee *et al*., have incorporated proton tautomerism^23^ and enhanced sampling methods to improve convergence.^24^

Discrete constant pH methods avoid non-physical intermediate charge states. These methods use MD simulations for conformational sampling, while sampling different discrete protonation states with periodic Monte Carlo (MC) steps interspersed throughout the MD trajectory.^14^–^18^ The methods employed in this article utilize the constant pH MD (CpHMD) method, originally developed by Mongan *et al*.,^14^ which uses generalized Born (GB) implicit solvent. Differences among these methods arise from choice of solvation model and protocols for updating protonation states within the simulation. Although these methods have achieved good results for small protein systems, they can be computationally expensive, and long convergence times have been reported for systems with multiple titration sites. In an attempt to overcome these issues of convergence, use of enhanced sampling methods coupled with constant pH MD, such as constant pH accelerated MD (CpHaMD)^25^ and constant pH replica-exchange MD (REX-CPHMD)^26^ have been investigated. Results from simulations employing these methods indicate the increased sampling provides improvement over the conventional method.

The previous paragraphs provide only a brief summary of the computational methods available for p*K*_a_ prediction. Further details of these and other methods can be found in the literature, and several reviews have been published.^27^–^29^ In this study, the successes and deficiencies of the CpHMD method have been investigated in the blind prediction of p*K*_a_ values of titratable residues of the WT and mutant forms of the Staphylococcal nuclease (SNase) enzyme based upon comparison to experimental results released after submission to the p*K*_a_ cooperative^30^ by García-Moreno and coworkers.^31^–^37^ Particular attention is paid to the differences in electrostatics and, consequently, acid/base properties of exterior and interior residues.

## THEORY

### Constant pH molecular dynamics—Theory background

CpHMD employs MD with GB implicit solvent.^14^ Within the simulation, the MD simulation is periodically halted, and a MC step is taken, randomly considering a titratable residue for change in protonation. The transition energy corresponding to this MC step is evaluated according to Eq. (1), which calculates p*K*_a_ with respect to a reference


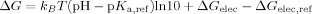
(1)

compound for the residue of interest. Reference compounds are the isolated titratable residues solvated in water (reference p*K*_a_ values are 3.8 for ASP, 4.3 for GLU, 6.8 for HIS, 9.6 for TYR, and 10.5 for LYS).^14^, ^38^, ^39^ In Eq. (1), *k*_B_ is the Boltzmann constant, *T* is the temperature, pH is the specified solvent pH, p*K*_a,ref_ is the p*K*_a_ of the reference compound, Δ*G*_elec_ is the electrostatic energy change for protonation state change of the titratable residue, and Δ*G*_elec,ref_ is the corresponding electrostatic transition energy for the reference compound. The same GB electrostatics employed in the MD is used for calculating this transition energy, with acceptance of the change in protonation determined by the Metropolis criterion. If the MC move is accepted, the protonation state of the residue will change to the new state, and MD is continued. If not, the simulation will continue with the residue remaining in the unchanged protonation state. CpHMD has been successfully applied in the p*K*_a_ prediction of titratable residues in the Hen Egg White Lysozyme (HEWL) enzyme.^14^

### Titration curve construction and p*K*_a_ calculation

The predicted p*K*_a_ values are calculated from performing CpHMD simulations over a range of solution pH values. Assuming the system is ergodic, we assume fractional protonation is given by the amount of time a particular titratable residue spends in its protonated state.^15^ Thus the fraction of deprotonated species, *s*, for a residue at a specific pH value can be used to predict the p*K*_a_ from a Hill plot [Eq. (2)].^16^, ^20^, ^22^, ^40^ Fits to this curve


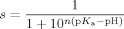
(2)

allow for estimation of both the p*K*_a_ value as a midpoint of titration, as well as the Hill coefficient, *n*, which describes the cooperativity of various sites with respect to titration.^41^ Illustrated in Reference 40, for example, the usefulness of the Hill equation resides in its ability to provide a good prediction of the midpoint p*K*_a_ value, even when the fit is inaccurate at the tails of the titration curve.^40^

## METHODS

### Test system: Staphylococcal nuclease

Staphylococcal nuclease (SNase) is a highly charged protein, which has generated difficulty in obtaining accurate structure-based p*K*_a_ predictions.^42^ The structures of the wild-type and mutant proteins of the SNase system are provided for this study by the lab of Garcia-Moreno *et al*., who measured the p*K*_a_ shifts of the titratable residues using NMR spectroscopy.^31^–^37^ Along with other computational groups, we have computed blind p*K*_a_ predictions for residues of wild-type SNase (PDB ID: 1STN or 1SNC), the SNase mutant Δ+PHS (PDB ID: 3bdc), and various mutants from the Δ+PHS parent protein (referred to as calculated results in this study). Δ+PHS is unique in that it is a hyperstable, acid-resistant SNase mutant with five substitutions (G50F, V51N, P117G, H124L, and S128A) and a deletion of residues 44–49.^31^, ^32^ Garcia-Moreno directed this effort, holding experimental p*K*_a_ determinations from those making predictions and picking residues of interest for p*K*_a_ prediction (hereafter referred to as experimental results in this study). In total, approximately 93 structures of the wild-type (WT) and mutant SNase have been provided.^30^ Owing to time constraints, however, our CpHMD p*K*_a_ predictions have not been carried out on the entire set of provided structures, the subset of which were studied and submitted as blind predictions shown in Table I.

**I:** Predicted and Experimental Values for Various Residues from the WT SNase, Δ+PHS, and Δ+PHS Mutant Proteins^30^,^31^

Protein	Residue	Experimental p*K*_a_	Predicted p*K*_a_	(Pred.−Exp) p*K*_a_ offset	(Pred.−Model) p*K*_a_ offset
WT	HIS8	6.52	5.67 ± 0.04	−0.85	−1.1
	HIS46	5.86	6.8 ± 0.3	0.7	0.0
	HIS121	5.30	7.0 ± 0.1	1.7	0.2
	HIS124	5.73	6.0 ± 0.1	0.3	−0.8
Δ+PHS	ASP19	2.21	4.1 ± 1.1	0.9	0.3
	ASP21	6.54	–	–	–
	ASP40	3.87	3.1 ± 0.1	−0.8	0.7
	ASP77	<2.2	3.6 ± 0.2	>1.1	−0.2
	ASP83	<2.2	2 ± 8	−	−2
	ASP95	2.16	3.6 ± 0.1	1.4	−0.2
	GLU10	2.82	4.4 ± 0.2	1.6	0.1
	GLU43	4.32	1 ± 5	−0.9	−3
	GLU52	3.93	4.3 ± 0.2	0.4	0.0
	GLU57	3.49	4.3 ± 0.1	0.8	0.0
	GLU67	3.76	4.39 ± 0.03	0.6	0.09
	GLU73	3.31	4.2 ± 0.1	0.9	−0.1
	GLU75	3.26	4.0 ± 0.1	0.7	−0.3
	GLU101	3.81	3.5 ± 0.2	−0.3	−0.8
	GLU122	3.89	3.8 ± 0.1	−0.1	−0.5
	GLU129	3.75	4.28 ± 0.04	0.6	−0.02
	GLU135	3.76	4.2 ± 0.1	0.4	−0.1
F34E	GLU34	7.30	5.9 ± 0.1	−1.4	1.6
F34K	LYS34	7.10	2 ± 5	−5	−3
G20D	ASP20	<4.0	2 ± 2	−2	−2
G20E	GLU20	<4.5	4.1 ± 0.3	–	−0.2
G20K	LYS20	>10.4	8.6 ± 0.2	<−1.8	−1.8
L25D	ASP25	6.80	4.8 ± 0.3	−2.0	1.0
L36D	ASP36	7.90	5 ± 3	−3	1
L37D	ASP37	<4.0	–	–	–
V23D	ASP23	6.8	3 ± 2	−4	−1
V23E	GLU23	7.1	6.4 ± 0.1	−0.7	2.1
V23K	LYS23	7.40	7.3 ± 0.6	−0.1	−3.1

The difference between experimental and model compound p*K*_a_ values for Δ+PHS mutants. Model compound p*K*_a_ values: 3.8 (ASP), 4.3 (GLU), 6.8 (HIS), and 10.4 (LYS).^14^,^38^,^39^

### CpHMD simulations

The standard CpHMD method has been implemented in AMBER10 molecular dynamics program. All simulations are conducted with the AMBER99SB force field^43^ and the GB solvent model igb=2,^44^–^46^ using a 30 Å cutoff value for nonbonded interactions and computation of effective Born radii calculations. Similar to experimental conditions, salt concentrations are set to either 0.1 or 1.0*M*. The SHAKE algorithm constrains all bonds involving hydrogen with a time step of 2 fs,^47^ and temperature is maintained at 300 K using the Berendsen temperature coupling method with a time constant of 2 ps.^48^ A period of 10 fs of MD separates the MC trials. With these parameters, a 10 ns CpHMD simulation takes approximately 72 h using 16 Xeon X5650 2.67GHz processors.

All simulations begin from the crystal structure coordinates of the WT and Δ+PHS SNase systems provided by Garcia-Moreno *et al*., from which specific titratable residues were chosen for the blind prediction study (Table I). For the blind predictions performed on SNase systems where a single ASP or GLU residue has been highlighted as the residue of interest, CpHMD simulations of 10 ns in length have been performed in the solution pH range 2.0–7.0 at 0.5 pH unit intervals, titrating only acidic residues. For these simulations, HIS residues are allowed to titrate from pH 4.5 to pH 7.0. In systems where a LYS or TYR is highlighted as the residue of interest, simulations have been carried out in the pH range 7–10.5, where HIS, LYS, and TYR residues are set to titrate. The exclusion of HIS residues from the most acidic simulations is justified, as the p*K*_a_ of the HIS reference is around 6–7.^39^ In most cases, it is safe to assume all ASP and GLU residues are deprotonated above pH 7 and all LYS and TYR are protonated below pH 7, allowing exclusion of these residues from titration in these respective pH regions. Models for the terminal residues have not yet been developed for this system, so these residues are set to their most likely protonation states at neutral pH, with the N-terminus protonated and the C-terminus deprotonated. All non-titrating residues are set to their expected protonation states.

### Simulations conducted after publishing of experimental results

To understand why some predictions fail to reproduce the experimental results, further CpHMD simulations have been conducted, as indicated in the proceeding sections. In particular, the pH range at which simulations were originally performed is extended to account for residues that deviate the most from their reference value. In cases where convergence has been determined to be problematic, extended simulations (>10 ns) do not appear to improve predictions (results not shown).

## RESULTS

### Titration curves

Titration curves are obtained from CpHMD simulations for 32 titratable residues of the WT, Δ+PHS, and mutant Δ+PHS SNase systems. The experimental p*K*_a_ values for the WT protein were published prior to the predictions, but those for the mutant proteins were withheld until blind predictions were made (Table I). From Eq. (2), p*K*_a_ values are calculated along with the standard errors of regression for curve fits to the Hill equation in Eq. (2) (Table I).^49^ In the instances of ASP21 and L37D, p*K*_a_ values cannot be computed due to the lack of transitions between protonated and deprotonated forms.

**Figure 1:**
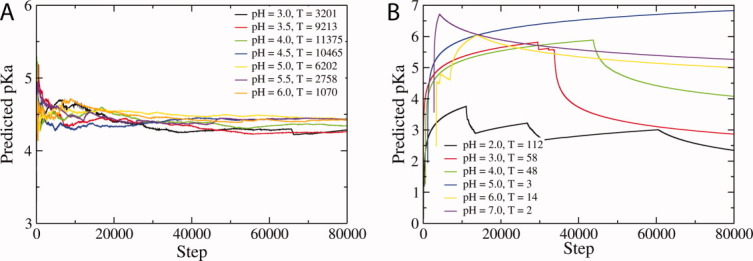
Plots of predicted p*K*_a_ over the duration of CpHMD simulations for (**a**) Δ+PHS GLU52 (experimental p*K*_a_ = 3.93)^31^ and (**b**) Δ+PHS L36D (experimental p*K*_a_ = 7.90).^35^ The number of protonation state transitions (T) are given in the figure legend for each system.

A representative plot of calculated p*K*_a_ over time is given in Figure 1 for both a surface residue for which the CpHMD predicts p*K*_a_ accurately (Δ+PHS, GLU52) and the Δ+PHS mutant L36D, for which the p*K*_a_ prediction of the interior residue ASP36 deviates by more than three p*K*_a_ units from the experimental result. In the former case, the p*K*_a_ converges rapidly, whereas ASP36 in Δ+PHS L36D is indicated to not achieve convergence over the duration of the simulations.

In assessing the convergence of a system, it is interesting to observe the trend in the number of transitions between protonated and deprotonated states as a function of pH. In systems that are well converged (e.g., Δ+PHS, GLU52), the greatest number of transitions between deprotonated and protonated states within the CpHMD scheme are found for the simulation conducted at a pH nearest to the calculated p*K*_a_ value. Simulations conducted at pH values far from the predicted p*K*_a_ encounter fewer transitions between protonation states, as is to be expected from the acceptance criteria defined in Eq. (1). This is not the case for certain systems (e.g., Δ+PHS L36D), where convergence is a problem. Thus the presence of a clear distribution of transitions across the different pH values simulated, peaked at the pH nearest the predicted p*K*_a_, may be an indicator of how well converged the system is.

### Experimental validation

**Figure 2:**
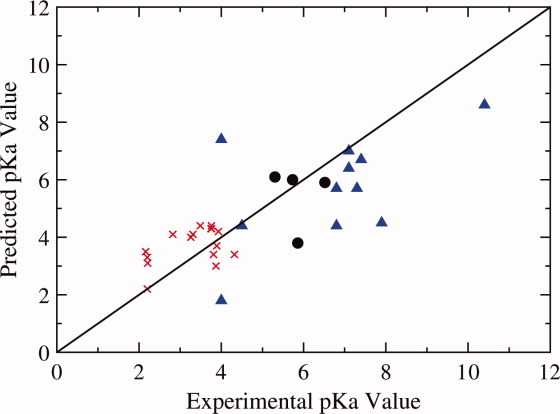
Plot of predicted versus experimental p*K*_a_ values for WT SNase (•), Δ+PHS (x, exterior residues), and Δ+PHS mutants (Δ, internal residues).^31^–^37^ The line y = x represents accurate prediction of the experimental p*K*_a_.

Following the blind predictions, García-Moreno and coworkers have released experimental results for comparison to predicted p*K*_a_ values (Table I).^30^ In summary, CpHMD simulations calculate the p*K*_a_ values of 17 residues to within 1 p*K*_a_ unit of the experimental value, 9 residues within 2 units, 1 residue within 3 units, and 2 residues within 4 units. Residues in the Δ+PHS protein chosen for analysis are surface residues. All of these residues remain solvent-exposed throughout the CpHMD simulation and are generally well predicted with respect to experiment (Table I). From Figure 2, it is clear that the predictions with the largest deviation from the experimental values are for Δ+PHS variants that have residues located within the hydrophobic interior of the protein (e.g., L37D, L36D). These residues have also been found experimentally to have the largest shifts in p*K*_a_ from their reference values (Table I). Errors with respect to the experimental p*K*_a_ are shown in Table I, with predictions within ranges of experimental p*K*_a_ considered to have zero error.

As described in Table I, CpHMD simulations have correctly predicted the experimental trends for the majority of these buried residues, three within 1 p*K*_a_ unit of the experimental result (G20E, V23E and V23K). The simulations predict the p*K*_a_ values of F34E/K, L25D, L36D, and V23E/K to be shifted from their model values in the direction of favoring the neutral residue at physiological pH; although, for some of these residues, the shift in the predicted p*K*_a_ is not as large as that found experimentally (Table I).

## DISCUSSION

### Residues with p*K*_a_ predictions greater than 1 p*K*_a_ unit from experimental

Since the release of experimental results, further simulations have been carried out to investigate why our methods predict p*K*_a_ values that deviate more than 1 p*K*_a_ unit from experimental results. For this article, we have chosen a selection of residues that illustrate problems with the application of the CpHMD method to these specific systems.

### Δ+PHS: ASP21

In the Δ+PHS mutant, the residue ASP21 is a notable exception to the good performance of CpHMD in predicting p*K*_a_ values of surface residues. This problem arises from a lack of transitions between protonated and deprotonated states. In this case, longer simulations fail to alleviate the problem, likely due to the existence of a strong, charged hydrogen bond interaction between ASP19 and ASP21 preventing changes in protonation state from occurring. Consistent with our results, García-Moreno and coworkers have needed to apply two-site binding isotherms to properly describe the experimental titration of these interacting residues and have also noted the difficulty in predicting the p*K*_a_ for ASP21 computationally.^31^ Similar problems arise in the simulation of L37D, indicating that sampling of protonation states is critical to the performance of the CpHMD method. Use of enhanced sampling techniques to allow the system to sample other protonation states may be necessary for accurate p*K*_a_ predictions in conventional simulations where strong interactions persist.

### Δ+PHS G20K

**Figure 3:**
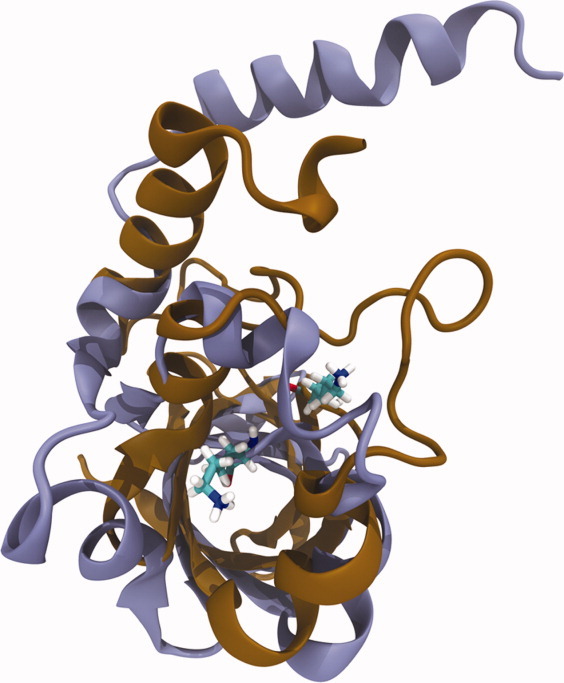
Conformational change encountered by the Δ+PHS G20K protein at the start (copper) and end (purple) of CpHMD simulation performed at pH 8.5.

For the mutant Δ+PHS G20K, the CpHMD method predicts a p*K*_a_ of 8.6, nearly two p*K*_a_ units lower than the experimental value (>10.4).^33^ This lysine residue sufficiently sampled protonation space, encountering more than 600 transitions over the duration of each simulation. The trajectories of these simulations incur large motions indicative of protein instability. The root mean square distances (RMSD) with respect to the starting structure for these simulations do not converge at any solution pH, largely influenced by the winding and helical motion of the last 20 residues of C-terminus (Fig. 3). To further probe this conformational change, we performed conventional MD simulations with set protonation states computed by the program PRO p*K*_a_^50^ for pH 7–10 at intervals of 0.5. The conventional MD simulations similarly suffer from protein instability near neutral pH (pH 7 and pH 8), although take longer to encounter it than CpHMD simulations. At higher pH, the terminal helix in the G20K protein does not incur the same motion observed at neutral pH and in CpHMD simulations. These simulations show the sensitivity of the G20K protein toward change in protonation state, and in order to achieve results closer to experiment with the CpHMD method, it may be necessary to spatially constrain the termini. These findings indicate that although increased sampling is desirable and may be achieved in certain systems, it is important that the correct conformational space is sampled to attain an accurate prediction of p*K*_a_.

In further probing the problems involving predicting the p*K*_a_ for G20K, it is noteworthy that other mutations at site 20 generate similar instabilities (e.g., G20D and G20E). Having an acidic residue at site 20, however, does not affect the p*K*_a_ prediction to the same extent. Visual analysis of trajectories for the G20D protein reveals that hydrogen bonds from Thr-29 persist throughout the simulations, likely lowering its p*K*_a_ (Table I). Similarly, G20E forms transient hydrogen bonds with Thr-29. All mutated residues at site 20 sample conformational space that is solvent-exposed, in addition to time spent buried in the protein interior. While this explains the propensities for G20D and G20E to exist in their charged states, it fails to explain the shift in p*K*_a_ for G20K.

### Δ+PHS F34E

**Figure 4:**
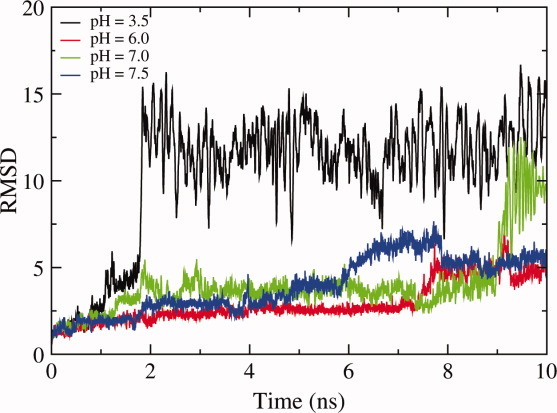
RMSD as a function of MD time step for the Δ+PHS mutant F34E protein at varying pH values.

CpHMD simulations performed on the Δ+PHS F34E mutant consistently obtain a predicted p*K*_a_ (5.7) lower than experiment (7.30).^32^ The stability of this particular mutant shows great sensitivity to the pH of the simulation, with large conformational changes occurring at acidic pH (Fig. 4). Nevertheless, the p*K*_a_ values calculated at neutral pH—closer to the p*K*_a_ of the residue—still underestimate the experimental p*K*_a_ despite undergoing a large number of transitions between protonated and deprotonated states. Upon visualization of this structure, it is notable that the carboxylate of GLU34 forms salt bridges with an adjacent arginine residue (ARG81), which causes this residue to favor its deprotonated state. The simulations may not sample enough conformational space owing to the persistence of this salt bridge, therefore leading to a predicted p*K*_a_ value lower than the experimental result. Enhanced sampling techniques may provide the means to allow the system to escape this GLU34-ARG81 salt bridge and give a more representative prediction of p*K*_a_.

### Δ+PHS L36D

The mutant L36D suffers from sampling problems, both of conformational and protonation space (Fig. 1). While at certain pH values CpHMD simulations correctly predict the p*K*_a_, which experimentally is found to be 7.90,^36^ there is no clear trend in p*K*_a_ prediction for simulations conducted at different levels of pH (Fig. 1). From visualization of the various trajectories, it is suggested that ASP36 may form a strong hydrogen bond with ASP21 in the MD simulations, stabilizing the deprotonated form. This scenario is seen at pH 4.5, where the simulation more accurately predicts a p*K*_a_ of 7.4. In other cases, ASP36 becomes buried in the hydrophobic interior of the protein, again leading to insufficient sampling of different protonation states. It is therefore likely that L36D needs to better sample conformational space in order to more effectively predict the p*K*_a_ of ASP36.

### Analysis of CpHMD performance

It is clear the CpHMD method performs better at predicting the p*K*_a_ values of solvent-exposed residues, which possess p*K*_a_ values closer to their reference compounds (Table I). This is evident from the calculation of the root mean square error (RMSE) of predicted p*K*_a_ values, measured against the experimental work of García-Moreno to quantify this result, showing that residues on the surface of Δ+PHS deviate from experiment with an RMSE of 1.23, whereas the RMSE for interior residues of the various Δ+PHS mutants is 2.42 (Table II).^31^ Most residues found at the surface of the protein encounter an increased number of transitions between protonated and deprotonated forms, and tend to converge relatively quickly (∼6–8 ns). The counterexample to this trend is ASP21, which likely fails to transition due to sampling problems derived from the persistence of its hydrogen bond with ASP19.

**II:** RMS Errors of Predicted p*K*_a_ Values Against Experimental Values for Residues Located in Different Regions of the Δ+PHS Protein (Exterior Residues) and Δ+PHS Mutants (Interior Residues)^31^

	Surface	Interior
All residues	1.23	2.42
Aspartates	1.22	2.59
Glutamates	1.23	0.90
Lysines	–	3.12

Errors are computed with zero error if the predicted p*K*_a_ falls within the bounds of experimental p*K*_a_ with limiting values. Residues that do not incur transitions (ASP21 and L37D) are omitted from this calculation.

The importance of selecting a suitable pH range for titration and difficulties in achieving proper sampling of conformational space are illustrated in some p*K*_a_ predictions of interior residues for the various Δ+PHS mutants. Given that many internal residues are found experimentally to have p*K*_a_ values shifted considerably from their reference p*K*_a_, it is thus important to set up simulations over a wide pH range to conduct the titration. For example, in the case of L36D, simulations were performed at acidic pH under the assumption that the p*K*_a_ of the aspartic acid would exist closer to its reference value of 3.8. In fact, experimental results show this residue to titrate at a p*K*_a_ of 7.80. The selection of the pH range is also important for the stability of the system when performing CpHMD simulations, as illustrated by Δ+PHS F34E.

Analyses of computed p*K*_a_ over time show internal residues to be far less converged compared to surface residues, making the prediction of accurate p*K*_a_ values more challenging. Although the CpHMD method applied in this study usually predicts the direction of the p*K*_a_ shift from the reference compounds correctly, there is still room for improvement in accurately predicting p*K*_a_ for internal residues.

Residues buried within the protein environment experience dielectric environments quite different from those at the surface of the protein, with their p*K*_a_ properties very susceptible to the nature of the residues in their vicinity and thus more difficult to treat computationally.^31^, ^42^ This difficulty is illustrated by the Δ+PHS L36D and F34E proteins, where strong hydrogen bonds or salt bridges involving these titratable residues affect their protonation equilibria. Simulations of L36D do not contain any transitions between deprotonated and protonated forms owing to the persistence of an interaction between the ASP36 and ASP21 residues. For residues such as this, the use of an enhanced sampling method, such as accelerated MD, may assist in the sampling of relevant conformations and thus protonation states. The requirement for increased sampling is also highlighted in instances where salt bridges persist throughout the simulation, as in the case of ARG81-GLU34 in the F34E mutant protein. The CpHMD method severely under-predicts the p*K*_a_ of this glutamate, suggesting it spends more time in its deprotonated form than experiment predicts.^31^ It is possible, although not proven in our studies, that the strength of salt bridges sampled in our CpHMD method is overestimated under the GB implicit solvation, thus leading to error in predicting protonation state.^51^

Although not specifically quantified in this study, errors likely exist in CpHMD simulations due to the use of implicit solvation and conventional (non-polarizable) force fields. With regards to implicit solvation, issues regarding global protein movements would likely be dampened by the presence of explicit solvent molecules. Despite this generally accepted point, we believe CpHMD simulations employing implicit solvation still merit further study due to the simplicity of protonation changes and transition energy calculations. With regards to force fields, polarizable force fields would likely better capture the sensitivity of neighboring groups to changes in the protonation state. The topic of force field effects on constant pH MD simulations is further investigated by others in this issue.^52^

Although there exist problems with both implicit solvation and conventional force fields, the CpHMD method has been successful in predicting the p*K*_a_ of a significant number of residues from the test set from García-Moreno. This study has highlighted areas that may add significant improvement in the p*K*_a_ prediction capability of the method, such as enhanced conformational sampling and implementation of an improved solvation model. Future work will focus on testing other solvation models and the implementation of different accelerated molecular dynamics techniques, with the goal of achieving better sampling of physically meaningful conformations and protonation states.
